# Emotional Movement Kinematics Guide Twelve‐Month‐Olds’ Visual, but Not Manual, Exploration

**DOI:** 10.1111/infa.70000

**Published:** 2025-01-22

**Authors:** Joanna M. Rutkowska, Julia Mermier, Marlene Meyer, Hermann Bulf, Chiara Turati, Sabine Hunnius

**Affiliations:** ^1^ Donders Institute for Brain, Cognition and Behavior Radboud University Nijmegen The Netherlands; ^2^ Department of Psychology Jacobs Center for Productive Youth Development University of Zurich Zurich Switzerland; ^3^ Department of Psychology University of Milano‐Bicocca Milan Italy; ^4^ NeuroMI Milan Center for Neuroscience Milan Italy

**Keywords:** emotion, emotion processing, exploration, kinematics, movement

## Abstract

The ability to recognize and act on others' emotions is crucial for navigating social interactions successfully and learning about the world. One way in which others' emotions are observable is through their movement kinematics. Movement information is available even at a distance or when an individual's face is not visible. Infants have been shown to be sensitive to emotions in movement kinematics of transporting actions, like moving an object from one to another place. However, it is still unknown whether they associate the manipulated object with the emotions contained in moving it, and whether they use this information to guide their own exploration of this object. In this study, 12‐month‐old infants watched actors transporting two toys with positive or negative emotional valence. Then, infants were given the possibility to interact with the same toys. We expected the infants to look at and touch the toy handled in a positive manner more, compared to the toy handled in a negative manner. Our results showed that infants looked at the positive toys more than at the negative toys, but that infants touched both toys for the same amount of time. Also, there was no difference in which toy they manually explored first.

## 1 Introduction

Emotions change the way we behave and interact with the world (Frijda 2010). The ability to recognize others' emotions is crucial for navigating our environment (e.g., Leppänen and Hietanen 2001). To recognize others' emotions, one can use various cues, such as people's facial expression (Langner et al. 2010), voice (Lassalle et al. 2019), and movement kinematics (Pollick et al. 2001). In the latter case, the emotions can be observed from body expressions and postures (Dael, Mortillaro, and Scherer 2012; de Gelder 2006), walking gait (Montepare, Goldstein, and Clausen 1987; Barliya et al. 2013), and even the differences in movement of everyday actions, such as drinking or knocking (Gross, Crane, and Fredrickson 2010; Pollick et al. 2001). Body movements have been proposed as an important source of emotion recognition that enables us to react quickly and adaptively (Edey et al. 2020; Roether et al. 2009; de Gelder 2006). This is because of the visual availability of the information in body movements even at a distance (as opposed to facial expressions; de Gelder 2009) and due to the rapid and partly automatic nature of motor simulation of others' movements (Edey et al. 2017, 2020). While emotion perception from faces and voices in infancy is well‐documented (e.g., Walker‐Andrews 1998; Datyner, Henry, and Richmond 2017), very little is known about emotion recognition from movement kinematics early in life. Only a handful of studies have investigated this process in infancy and their results paint a complex picture of the development of emotion recognition from movement kinematics, and its influence on infants' behavior.

After only a few days of life, infants are already able to discriminate human movements from other movement patterns (Simion, Regolin, and Bulf 2008), and after a few months, they readily attend to adults' movements (Fausey, Jayaraman, and Smith 2016). Therefore, it is not surprising that they are sensitive to various features of movement kinematics early on, such as goal‐directedness or emotional information. For instance, the ability to discriminate emotions when expressed by whole body movements has been shown in infancy (e.g. Heck et al. 2018; Zieber et al. 2014). These were not everyday movements carrying emotional information, but rather whole body expressions aimed at communicating an emotion. For instance, happiness is portrayed as jumping up and down with raised arms, and anger is portrayed as raising the arms and shaking the fists. However, in daily life, other people rarely display these whole body expressions. Instead, their emotions can be visible in the variability of their everyday movements (e.g. Pollick et al. 2001; Gross, Crane, and Fredrickson 2012). Only three studies examined infants' emotion perception from everyday movements. Ogren and colleagues (2019) presented 10‐ to 20‐month‐old infants with point‐light displays of emotional walking movements carried out by adults. They used a preferential looking paradigm to examine whether infants distinguished between walking movements of different emotions. The results suggested that although the infants showed evidence of discrimination between emotions in walking, this effect was likely driven by low‐level motion preferences. Moreover, Addabbo and colleagues (2020) showed that 11‐month‐old infants are sensitive to emotions conveyed in movement kinematics of an arm movement. Infants were presented with an actor picking up a toy and moving it to a box with happy or angry movements. Importantly, only the torso and the arm of the actor, but not their face, were visible in the stimulus movies. While the infants were watching, the activity of their facial muscles was recorded with facial electromyography (EMG). The infants showed an emotional facial response matching the emotional valence of the stimuli they were watching. That is, during the action performed with happy kinematics, the activity in the muscle most responsible for expressing happiness increased and the activity in the muscle most responsible for expressing anger decreased, and vice versa for the action performed with angry kinematics. Schröer and colleagues (2022) used the same paradigm with 12‐ to 13‐month‐olds to investigate which mechanisms might underlie the emergence of the capacity to recognize others' emotions through kinematics. They found that negative expressivity in infants' primary caregivers was positively related to their sensitivity to angry kinematics. Thus, infants' observational experience with others' displays of emotion likely plays a role in the development of the ability to recognize others' emotions from their movement kinematics.

Related research shows that infants use others' behavior, including their facial and vocal expressions, to guide their own behavior and environment exploration (Brosseau‐Liard and Poulin‐Dubois 2014; Carpenter, Akhtar, and Tomasello 1998; Vaish, Grossmann, and Woodward 2008; Hunnius, Bekkering, and Cillessen 2009). This phenomenon has been extensively studied under the umbrella term of “social referencing.” Infants display this behavior when they encounter new environments or ambiguous situations. For example, infants use others' emotions to guide their behavior when confronted with a visual cliff (e.g., Sorce et al. 1985; Vaish and Striano 2004), novel toys (e.g., Hornik, Risenhoover and Gunnar 1987), or strangers (e.g., Feinman and Lewis 1983). This is not only true for emotional cues given directly by the primary caregivers, but also for the cues given by the unfamiliar adults (e.g. Repacholi 1998; Repacholi et al. 2016; Reschke, Walle, and Dukes 2020). For instance, Mumme and Fernald (2003) presented infants with videos of actors attending to objects and expressing positive, negative, or neutral vocal and facial emotional signals. Then, the infants could play with the same objects as shown in the stimuli. Twelve‐month‐olds, but not 10‐month‐olds, avoided playing with an object previously associated with negative emotions, choosing more frequently to play with other objects instead. Thus, their object exploration was influenced by the combined vocal and facial emotional displays. Infants can also use others' emotions to guide their action imitation. Repacholi and colleagues (2016) showed that 15‐month‐old infants were hesitant to perform an action when an adult nearby them previously reacted angrily (in facial and vocal emotional expression) when someone else performed it. The above‐mentioned studies used a combination of several emotional cues, and none of them included movement kinematics. If emotional movement detection is to enable a quick and adaptive response, one can hypothesize that it be able to modulate infants' behavior, even as the sole emotional cue. To sum up, although infants can use emotional information to guide their visual and manual behavior, it is still unknown whether they can use movement kinematics to do so, and whether they can rely on it as the only available emotional cue.

In this study, we investigated whether infants could use emotional information in movement kinematics to guide their object visual and manual exploration. We tested infants of 1 year of age, since children at this age have been shown to be sensitive to emotional information in movement kinematics (Addabbo et al. 2020; Schröer et al. 2022) and to use emotional information from other sources to guide their object exploration (Mumme and Fernald 2003). Infants were first presented with videos of actors transporting two toys, one with positive emotional valence, and one with negative emotional valence. Then, they were given the possibility to interact with the same toys themselves. If the infants detected the emotional information in movement kinematics and used it to guide their exploration, we expected them to look at and to touch more the toy previously moved with positive emotional kinematics, compared to the toy previously moved with negative kinematics. In addition, we expected them to first touch the toy previously moved with positive emotional valence compared to the other toy.

## 2 Methods

### 2.1 Participants

Fifty‐nine 12‐month‐old infants participated in the study. This sample size was based on a power analysis performed with G*Power (Faul et al. 2007) for a one‐tailed binomial test with power greater than 0.85, Hedges' *g* = 0.2, *α* = 0.05, which indicated a minimal sample size of 44. The one‐tailed *t*‐test was chosen for the power analysis due to the clear directionality of the hypotheses. To increase the statistical power needed to detect a small effect size, the researchers continued to recruit participants after the minimal sample size had been reached until the end of the internship period of the student who assisted with testing. Five infants were excluded from the analysis due to parental interference on both trials (*n* = 1), not watching at least four stimulus videos in each trial (*n* = 2), or experimenter error (*n* = 2). Thus, the final sample consisted of 54 infants (25 girls and 29 boys, mean age *M* = 12.4 months, SD = 0.43, range: 11.7–13.5 months). All participants were recruited from a database of families who signed up to participate in research at a middle‐sized city in the Netherlands. This study was conducted according to the guidelines laid down in the Declaration of Helsinki, with written informed consent obtained from a parent or a guardian for each child before any assessment or data collection. All procedures involving human subjects in this study were approved by the local research ethics board (ethical approval code: ECSW2016‐0905‐396). Families could choose to receive either a children's book or 10 euros as a compensation for their time.

### 2.2 Stimuli

#### 2.2.1 Toys

**FIGURE 1:**
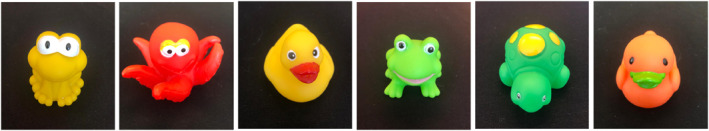
Pictures of the toys used in the study in stimulus and behavioral testing phases.

Infants were presented with stimulus videos showing six small toys: a green frog, a red octopus, a green turtle, a yellow duck, a yellow frog, and an orange duck (see Figure 1). These toys were also used in the behavioral testing phase during which the infants got to interact with them. They were bath toys made of rubber to allow for disinfection between testing sessions. The toys were also chosen to be easily distinguishable from each other, colorful and big enough to attract infants' attention, and small enough so that infants could easily pick them up with one hand.

#### 2.2.2 Video Stimuli

**FIGURE 2:**
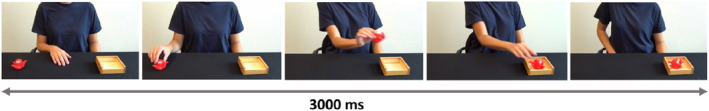
Series of frames from an example stimulus video.

The video stimuli consisted of 8 unique videos of an actor reaching for a toy and putting it in a wooden box using movement kinematics with a negative (*n* = 4) or positive (*n* = 4) emotional valence (see Figure 2; Rutkowska et al. 2024). The videos were recorded from a frontal point of view against a unicolor background, in stable lighting conditions, and the actors wore the same color t‐shirt. There were three different actors, all female. The actors were sitting behind the table, and thus were visible from the waist up, but their face was not visible on the video, so no facial emotional cues were available. Each video lasted exactly 3 s, and the movement was always carried out from left to right (see Figure 2). The six different toys were used as the moved object in the videos, and two toys appeared in movements with both positive and negative kinematics. Each participant was randomly allocated to watch 4 videos (2 in each trial), containing 4 different toys, in one of 4 pre‐determined orders (see Supporting Information S1: Table S1).

The video stimuli were created for this study, and validated in a study with adults (*n* = 28). Adults watched 77 videos of unique movements, and judged the emotional valence of the video (positive or negative), rated it on a Likert scale from negative (0) to positive (6), and chose a label for the emotion in the video. Eight videos were selected based on the following criteria: participant accuracy higher than 0.9 on the valence discrimination task, and the most frequently chosen label congruence with the emotional valence (“happiness” for positive, “disgust” or “fear” for negative). In addition, the videos were selected to allow for two sets of trials, and each trial set featuring different toys. The mean positive/negative discrimination accuracy of the chosen 8 video stimuli was 0.95. The mean rating of videos on the Likert scale was 3.61 for videos with positive emotional valence, and 1.54 for videos with negative emotional valence. The most common labels were: “happiness” for all the positive videos, “fear” and “disgust” for two of the negative videos each. For more information about the stimuli creation and validation, see Supporting Information S1.

### 2.3 Procedure

**FIGURE 3:**
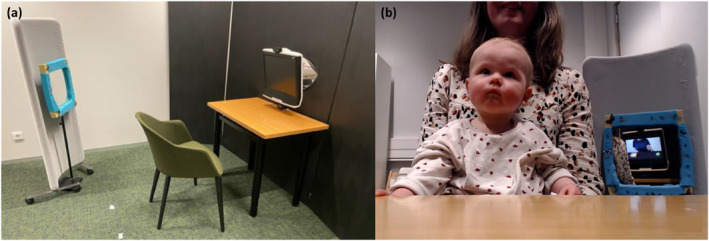
(a) Side view of the experimental set up with a chair for parents and infants to sit, a table with a mounted webcam under the stimuli presentation screen with built‐in eye tracker, and a mirror that enabled recording of the screen during the trial. (b) The view from the webcam during stimulus presentation.

Upon arrival at the laboratory, caregivers were informed about the general study procedure and asked to give written consent for their child to take part in the study. Then, they were asked to sit on a chair in front of a table and a screen with their child on their lap (see Figure 3). They were instructed not to watch the videos themselves and not to interfere with their child's behavior or speak during the study, unless they wanted to stop it. Thus, during the experiment, the infant participant sat on their caregiver's lap behind a 50 × 80 cm table, in front of a Tobii T120 eye‐tracker (17″ TFT monitor, 1.280 × 1.024 pixels, Tobii AB) at a viewing distance of about 60 cm from the eye‐tracker screen that was used for stimulus presentation with Tobii Studio (version 3.0.9.425, Professional edition, Tobii AB) software. A webcam was installed in the middle of the edge of the table on the opposite side from the participant, approximately 47 cm away from them. The webcam was used to record the entire experiment for offline coding of infants' behavior during the testing phase, and of infants' attention to stimuli during the stimulus presentation phase in case the eye‐tracking did not work. It captured the table, the child, the parent, and a mirror positioned behind them that enabled the researchers to see the contents of the screen on the webcam recording.

**FIGURE 4:**
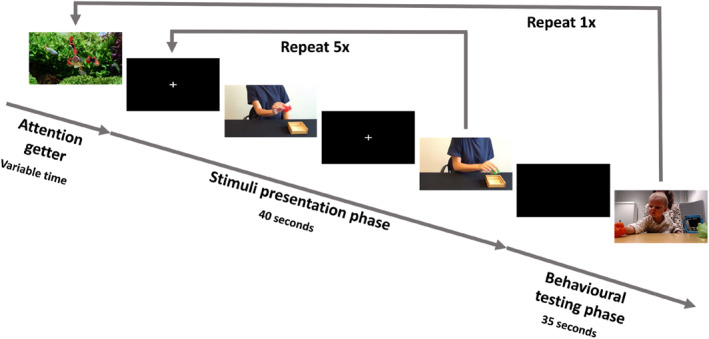
The figure illustrates the sequence of an example trial, including still images of the attention getter, the stimulus videos and a recording from the study showing a child grasping a toy.

The experiment started with the eye‐tracker calibration that involved a presentation of a moving toy image in five different positions on the screen (5‐point calibration). After successful calibration, the experimenter went to the back of the room (behind the infant) and the first trial started. In case of repeated incorrect calibration, the stimuli were presented, but no eye‐tracking data was collected (*n* = 1). There were two trials in the study. Each trial consisted of an attention getter, stimulus presentation phase, and behavioral testing phase, in that exact order (see Figure 4). The attention getter was played until the child was looking at the screen (determined by the second experimenter via live video‐feed). The stimulus presentation phase lasted 40 s and started with 1 s of fixation cross, followed by one video with positive or negative valence, another 1 s of fixation cross and a video with the opposite valence. In each trial, the videos featured one actor and two toys, but the actor and toys changed between trials to avoid any carry‐over effects. The color and the shape of the toys were different within each pair to allow for easy identification (see Supporting Information S1 for more information). Within each trial, the presentation of the two videos was repeated five times, which means the child was shown an actor moving each toy with positively or negatively valenced kinematics five times in each trial.

After stimulus presentation, a black screen appeared and the behavioral testing phase followed. The behavioral testing phase commenced with the experimenter placing the two previously shown toys in two pre‐determined spots on the table, at approximately 33 cm distance from each other and 16 cm distance from the edge of the table where the infant was positioned. This distance between the toys was determined during piloting and chosen to maximize the chances of the infant reaching for only one of them at a time and thus to encourage them to choose between them. At first, the toys were carried and placed behind a paper box that obstructed them from the infant's view. When the toys were in the right position and facing the infant, the experimenter said “Een, twee, hoppakee” (English: “One, two, here we go”), quickly removed the box and stepped aside. From that moment, the infant was allowed to play with the toys for up to 35 s, or until one of the toys was out of reach (e.g., when it fell on the ground). We used a 35‐s time window following Mumme and Fernald's (2003) paradigm. They used a 30‐s play period to which we added 5 s because piloting showed that infants needed about 5 s to orient to the toys following the experimenter leaving. After the trial was completed, the attention getter was played, and the experimenter retrieved the toys from the table or the infant. The second trial started immediately after, as soon as the child settled down again, and looked back at the screen. After the second trial was completed, the experiment was finished.

### 2.4 Preprocessing

The eye‐tracking data of the video presentation phase was exported to MATLAB‐compatible files and analyzed with a custom script to determine whether infants could be included in the analysis, that is, whether they watched at least 500 ms of at least 2 positive and 2 negative toy stimulus videos during the stimuli presentation phase. For infants who had missing or insufficient eye‐tracking data (*n* = 21) during the stimuli presentation phase, a trained observer looked at the videos of children watching the stimuli using ELAN (Version 6.2, 2021) and noted whether they watched at least 500 ms of each video in an excel sheet (video watched/video not watched). A second trained observer coded the videos from 5 children (24% of the children with insufficient eye‐tracking data) using the same procedure. The inter‐observer reliability was computed using Cohen's Kappa for binary categories (video watched vs. not watched). The kappa value was 0.8, indicating substantial agreement between coders (Landis and Koch 1977). We had no research questions related to children's looking behavior whilst watching the stimuli, and we did not hypothesize any differences or a lack thereof. Thus, these looking data were used only to determine whether the inclusion criteria were met, but they were not statistically analyzed.

During the behavioral testing phase, infant behavior was coded frame‐by‐frame from video using ELAN (Version 6.2, 2021) by a trained observer for looking at a toy, touching a toy, and parental interference from the start of the trial (i.e., when the box obscuring the toys is removed from view) till the end of the trial (i.e., 35 s from the start or when one of the toys is unavailable, e.g. if it fell off the table, or when the parent interferes). The infant was coded as looking at the toy when the direction of their gaze, as indicated by visible eye movements, was clearly directed towards the toy. In cases where the infant's eyes were not visible during a portion of the video (e.g. due to blinking or a hand movement), the infant was still coded as looking towards the toy if this occurred within a continuous sequence where their gaze was observed on the toy before and after the occlusion. This approach ensured that the coding captured the continuity of gaze behavior. This occurred mostly due to children closing their eyes, and did not occur frequently. The infant was coded as touching the toy when it was clear that their hand was making physical contact with the toy. Parental interference was coded when the parents verbally encouraged the infants to touch any toy or physically moved their arms or hands to touch any toys. A second trained observer coded the videos from 11 children (20% of the sample included in the analysis) using the same procedure. The inter‐observer reliability was calculated on the coded durations separately for the looks and the touches using the Intraclass Correlation Coefficient (ICC; Shrout and Fleiss 1979). The ICC(2,1) for the looking duration was 0.96 (95% CI: 0.92–0.98) indicating excellent reliability across coders (Cicchetti 1994). The ICC(2,1) for the touching duration was 0.97 (95% CI: 0.95–0.98) indicating excellent reliability across coders.

The data from video coding were then exported to further analyze it with Python and pre‐processed with a custom script. For descriptive statistics purposes, trial duration and the duration of looking and touching for the positive and negative toy, were extracted. For statistical analysis, three variables were calculated: first touch, looking proportion and touching proportion. *First touch* was obtained by taking the emotional valence of the toy the infant first touched during each trial, if they touched any toys that trial. *Looking proportion* was calculated for each toy for each trial by dividing the time of looking at the toy by the total time spent looking at any toy during the trial. Similarly, *touching proportion* was calculated for each toy for each trial by dividing the time spent touching the toy by the total time spent touching any toy during the trial. Thus, the possible values of both proportion measures ranged between 0 and 1. **Importantly**, **it was possible for the infant to look at two toys (with positive and negative valence) or touch the two toys (with positive and negative valence) at once,** for example **when they grabbed them both and put them close together. Therefore, the proportions of looking at and touching the toys with negative and positive valence are independent of each other and do not necessarily sum up to 1**. We decided to calculate the dependent variables as proportions of total time spent looking or touching to account for the difference in trial length between children caused by parental interference or children dropping the toys on the floor (in both cases, trials finished early), and for the individual differences between how much time children spent looking at and touching the toys out of overall trial time. The looking proportion measured visual exploration, and the first touch and the touching proportion measured manual exploration.

The data from behavioral trials shorter than 10 s, and from trials in which infants did not watch enough of the stimuli or their parents interfered during a trial, were excluded from statistical analysis (*n* = 2, 1.85% of total trials). In addition, the data from trials including the yellow duck as one of the toys were also excluded as several parents reported having the same toy at home.

### 2.5 Analysis

We used Bayesian generalized linear mixed models (GLMMs) to analyze whether the children looked at or touched the toy previously moved with positive valence more, compared to the toy previously moved with negative valence in two test trials. We used that approach, as opposed to more traditional approaches (e.g. *t*‐test against chance) for several reasons. Firstly, the children had unconstrained access to both toys during the behavioral testing phase, and thus could touch the positive and the negative toys at the same time. This reflects a real‐world situation where children can explore their environment, several objects at a time, if they want to. However, this meant that testing whether the children touched the positive toy for more than 50% of the time would not show that they preferred that toy over the negative toy, as the negative toy could have also been touched for over 50% of the time. In other words, it would not test for children's preference. Secondly, because our data were expressed as proportions, the values ranged between 0 and 1, and they did not always adhere to the normality assumption required by the *t*‐test. For instance, the proportion of touching had many values at both ends (0 and 1). Using a GLMM allowed us to take the data distribution into account without violating any assumptions. For the proportion of touching, we specified the zero‐one‐inflated beta family, which accounts for the continuous nature of the proportions, while accommodating the excess of extreme values at 0 and 1, which provided us with an accurate fit for our data. Thirdly, a *t*‐test assumes independent observations, so we would have needed to conduct separate *t*‐tests for each trial or averaged the values for each child. Using a GLMM enabled us to account for the non‐independence of the data by including the subject as a random effect, and to analyze both trials at once without violating any assumptions or averaging any data. Finally, we opted for a Bayesian GLMM instead of the frequentist approach due to the many additional advantages offered by the Bayesian approach, such using prior expectations (priors) to stabilize the estimate of model parameters, and the quantification of the strength of evidence for the alternative hypothesis, instead of a binary decision on whether or not to reject the null hypothesis, offered by the frequentist approaches.

To test how the emotional valence of the toy affected the looking and touching proportions, two Bayesian generalized linear mixed models (GLMM) were fitted using the *brms* package in R with a default prior (Makowski, Ben‐Shachar, and Lüdecke 2019a). The looking model predicted the proportion of looking towards each toy from its emotional valence (positive/negative) and trial (1/2) with subject as a random effect. Similarly, the touching model predicted the proportion of touching each toy from its emotional valence (positive/negative) and trial (1/2) with subject as a random effect. We used the default Region of Practical Equivalence (ROPE) values available in the *brms* package. The results of the GLMMs are reported in accordance with Makowski et al. (2019b) recommendations. For the interpretation of the indices of effect existence, please see Makowski, Ben‐Shachar, and Lüdecke (2019a). To test whether the proportion of infants who touched the positive toy first was different from chance (50%), a two‐tailed Bayesian binomial test was performed in JASP (Version 0.14.1.0) for each test trial with a uniform prior (Lee 2004). In addition, the relationship between infants' looking and touching is analyzed in Supporting Information S1. The data (excluding video), and the scripts used for analysis and to generate figures, are available online (Rutkowska et al. 2024).

## 3 Results

### 3.1 Descriptive Statistics

#### 3.1.1 Trial Duration

**FIGURE 5:**
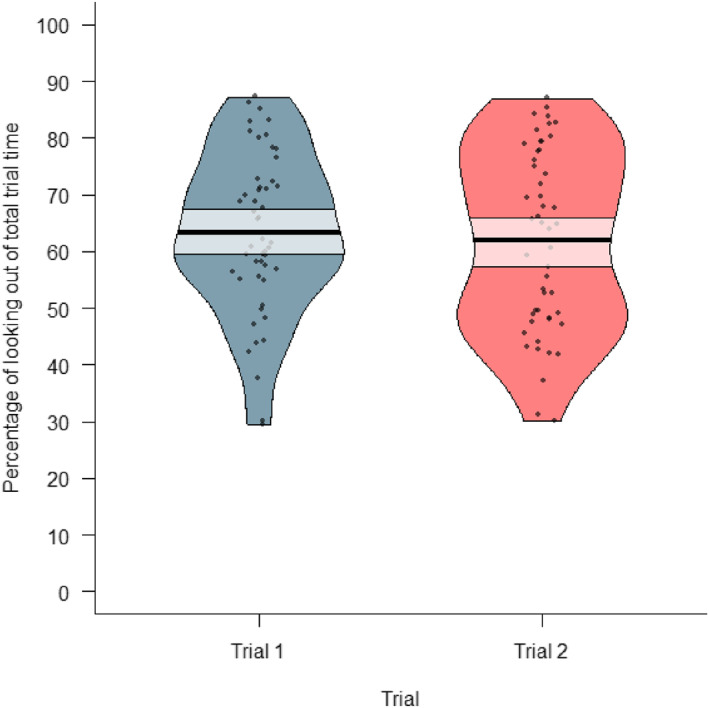
A pirate plot of the percentage of looking time out of total trial time [%] during trial 1 and trial 2; each dot represents a data point, the bold black horizontal line represents the mean of each condition, and the semi‐transparent box shows the 89% highest density interval (HDI) around the mean.

The mean trial time across both test trials was about 32 s (*M* = 31,698 ms, SEM = 739). More specifically, it was about 32 s (*M* = 31,906 ms, SEM = 946) in the first trial, and about 31 s (*M* = 31,490 ms, SEM = 1144; see Figure 5) in the second trial. Trials that took less than 10 s were excluded from all statistical analyses (*n* = 5).

#### 3.1.2 Looking Duration

The participants looked at a toy for an average of about 63% of the trial duration (*M* = 62.6%, SEM = 1.5) across both test trials, and 63% in the first trial (*M* = 63.3%, SEM = 1.93), and 62% in the second trial (*M* = 62.3%, SEM = 2.18; see Figure 5).

#### 3.1.3 Touching Duration

**FIGURE 6:**
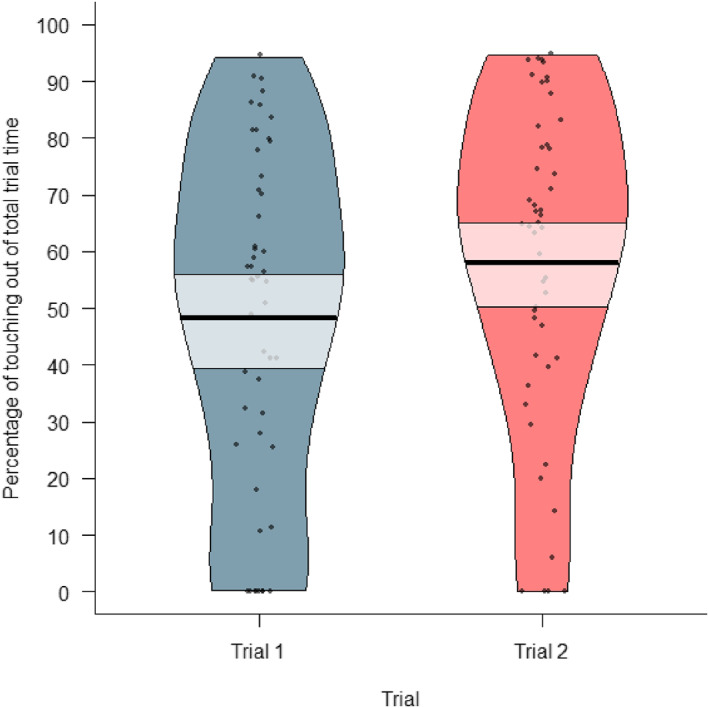
A pirate plot of the percentage of touching out of total trial time during trial 1 and trial 2; each dot represents a data point, the bold black horizontal line represents the mean of each condition, and the semi‐transparent box shows the 89% highest density interval (HDI) around the mean. Some participants did not touch any toy during a trial (0%).

The participants touched a toy for an average of about 53% of the total trial duration (*M* = 53.1%, SEM = 2.94) across both test trials, and 49% in the first trial (*M* = 49.5%, SEM = 4.15), and 57% in the second trial (*M* = 57.2%, SEM = 4.02; see Figure 6).

### 3.2 Proportion of Looking

**FIGURE 7:**
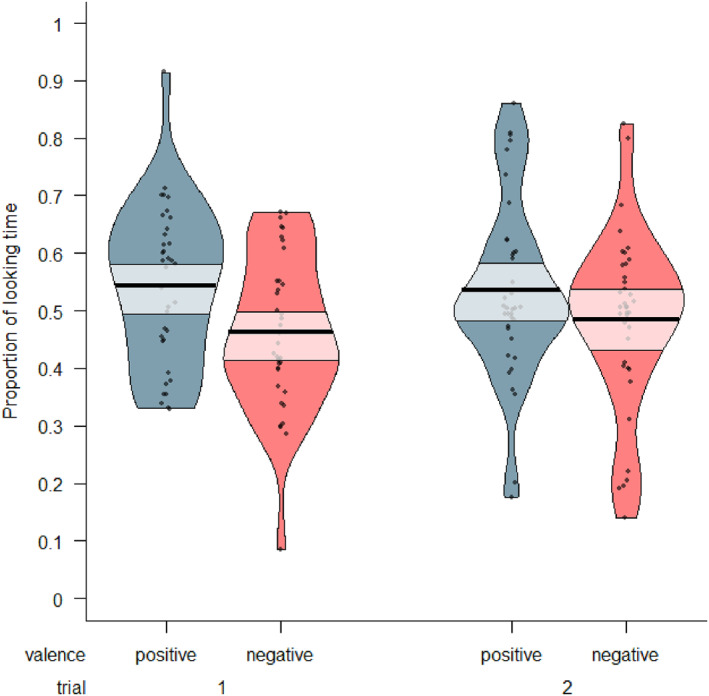
A pirate plot of the proportion of looking at positive and negative affect toys out of the total looking time during the trial; each dot represents a data point, the bold black horizontal line represents the mean of each condition, and the semi‐transparent box shows the 89% highest density interval (HDI) around the mean. Please note: a value of 1 means the participant looked at the toy for 100% of the time they looked at any toy during a trial, not of the total trial time.

To determine whether children looked more at the toys previously moved with either positive or negative emotional valence, we conducted a Bayesian Generalized Linear Mixed Model (GLMM). This model predicted the proportion of time children spent looking at a toy based on its emotional valence (positive or negative) and the trial (1 or 2), with individual differences accounted for by including subject as a random effect. The results indicated that children less likely to look at the toys associated with a negative emotional valence, compared to those with a positive valence. Specifically, there was a 99.8% probability that the effect of negative valence on the looking proportion was negative (Median = −0.07, 89% CI [‐0.10, −0.03]). This effect can be considered practically significant [0% in Region of Practical Equivalence (ROPE)] and medium in size (Standardized Median = −0.45). This means that infants spent less time looking at the toys associated with negative emotional valence. It was uncertain whether children's looking proportions differed between the two trials, as the effect of trial number on children's proportion of looking towards the toys had a 61.1% probability of being positive (Median = 0.006, 89% CI [‐0.03, 0.05]), and it was of undecided practical significance [52% in ROPE]. Thus, we found convincing evidence that children looked at the toy with positive valence for longer than at the toy with negative valence (see Figure 7). We did not find evidence that their looking changed between the two trials. For trace and density plots, see Supporting Information S1.

### 3.3 Proportion of Touching

**FIGURE 8:**
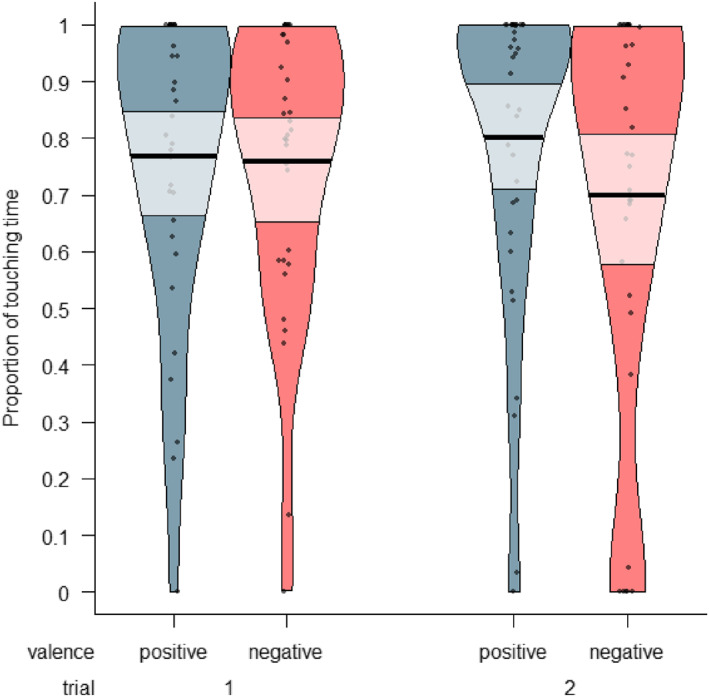
A pirate plot of the proportion of touching positive and negative affect toys out of the total touching time during the trial; each dot represents a data point, the bold black horizontal line represents the mean of each condition, and the semi‐transparent box shows the 89% highest density interval (HDI) around the mean. A value of 1 means the toy was touched for 100% of the time when any toy was touched during a trial, not of the total trial time.

To determine whether children touched more the toys previously moved with either positive or negative emotional valence, we conducted a Bayesian Generalized Linear Mixed Model (GLMM). This model predicted the proportion of time children spent touching a toy based on its emotional valence (positive or negative) and the trial (1 or 2), with individual differences accounted for by including subject as a random effect. The results indicated no convincing evidence that children's touching proportions differed between the toys, as there was a 65.1% probability that the effect of negative valence on the touching proportion was negative (Median = −0.08, 89% CI [‐0.42, 0.26]). Thus, it is uncertain whether children's proportion of touching was lower for the toys with negative valence compared to the toys with positive valence, and this effect can be considered of undecided practical significance [63.6% in ROPE]. It was uncertain whether children's looking proportions differed between the two trials, as the effect of trial number on children's proportion of looking towards the toys had a probability of 78.6% of being positive (Median = 0.18 89% CI [‐0.18, 0.53]), and it was of undecided practical significance [50.7% in ROPE]. Thus, we found convincing evidence that children looked at the toy with positive valence for longer than at the toy with negative valence (see Figure 8). Thus, we did not find convincing evidence that the children differed in how much they touched the toys with positive and negative valence (see Figure 8), or that their touching differed between the two trials. For trace and density plots, see Supporting Information S1.

### 3.4 First Touch

**FIGURE 9:**
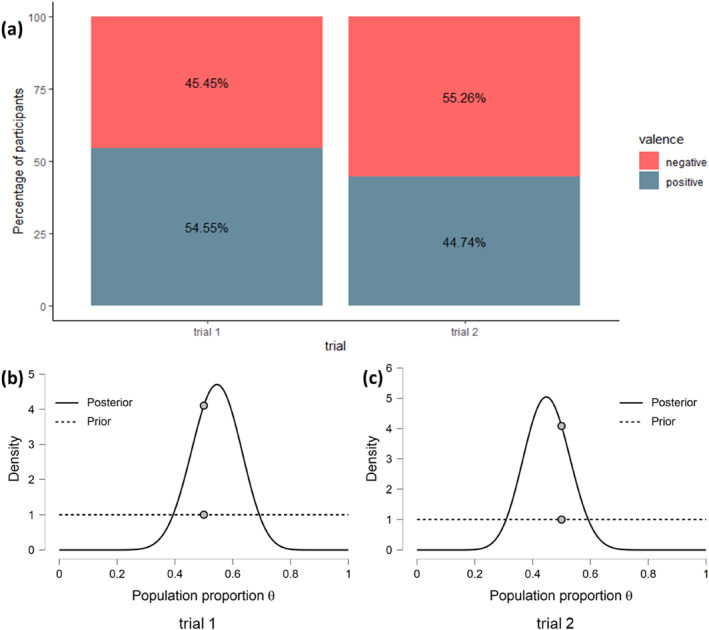
(a) A stacked bar plot of participants' first touch towards positively and negatively valenced toys on two test trials with percentages (%) of each choice. (b) The prior and posterior distribution of the population proportion *θ* for the Bayesian binomial test on the proportion of the first touch to positive toy difference from chance level (50%) for the first test trial the plot shows the estimate of the population proportion *θ* after updating prior knowledge with data. The gray dots indicate the density values of the prior and posterior distributions at test value. The dot being higher on the posterior than on the prior distribution shows evidence for the null hypothesis. (c) Analogously to the posterior distribution of the first trial, the posterior distribution for the second test trial is reflecting the evidence for the null hypothesis as the distance between the gray dots.

Two two‐tailed Bayesian binomial tests revealed moderate evidence that the proportion of the first touch to the positive toy was not different from chance in either trial 1 (proportion: 55%, BF01 = 4.11, 89% CI [0.39, 0.69]) or trial 2 (proportion: 45%, BF01 = 4.08, 89% CI [0.31, 0.59]). This means that the pattern in our data is 4 times more likely under the null hypothesis than the alternative hypothesis. Thus, we found support for the null hypothesis that participants did not first touch the positive toy above the chance level (see Figure 9).

## 4 Discussion

This study used a behavioral paradigm to examine whether 12‐month‐old infants use emotional information in the way others move (i.e. in movement kinematics) to guide their own visual and manual object exploration. The infants first watched videos of actors transporting a toy with positive or negative movement kinematics, and then they were given a chance to explore the same toys themselves. We made three hypotheses about infants' visual and manual exploration. We expected them to (1) look longer at, (2) touch more, and (3) touch first, the toy they had observed being handled in a positive emotional manner, compared to the other toy.

As hypothesized, infants looked longer at the toys that had been moved with positive emotional valence than with negative emotional valence. This suggests that infants perceived the differences in the emotional valence encoded in actors' movement kinematics. It also shows they linked the emotional valence to the object, since the differences in looking arose in the presence of the toys only, after the stimuli presentation was over and the actor was not visible on the screen. Moreover, the direction of the difference, that is looking longer at the toy associated with positive rather than negative emotions, was in line with findings based on infants' processing of facial emotional expressions. Thus, not only did the infants detect the difference in emotional valence in movement kinematics, but the direction of the effect suggests that they also extracted the emotional information and took it into consideration to guide their visual exploration. Contrary to our a priori hypotheses, we did not find differences in infants' manual exploration based on the emotional content in the movements. Infants did not touch any toy longer than the other, and they were not more likely to touch any of the toys first. Together with the looking time findings, this shows that the 12‐month‐old infants detected the emotional information in others' movements, and used this information to guide their visual, but not their manual, object exploration. In addition, in our supplementary analyses (see Supporting Information S1), we also found no relationship between infants' looking and touching proportions, suggesting that they were likely guided by different processes.

Although unexpected, the contrasting results between looking and touching measures are in line with some previous findings in the area of social development. Several studies found that children in the first 5 years of their lives demonstrate sensitivity to social information in their looking patterns, but fail to act on it (e.g. Nilsen et al. 2008; Brezack, Meyer, and Woodward 2021). For instance, Berman and colleagues (2016) used an emotion matching task to show that 3‐year‐olds looked more towards a facial expression matching the vocal emotional cue they were hearing, but they were not more likely to point towards it when asked. In contrast, 5‐year‐olds showed emotion matching both in their gaze and when asked to point. These differences in children's performance might arise from insufficient executive function skills, such as inhibitory control (Nilsen and Graham 2012), as infants in the current study did not use the emotional information to inhibit playing behavior with the toy associated with the negative emotion. However, we tested the participants at the same age as Mumme and Fernald (2003), who showed that at 12 months of age, infants are able to use emotional information in their play behavior, so infants in our study should not have been limited by cognitive resources. One key difference between the studies was the source of emotion information: whereas Mumme and Fernald (2003) used an actress expressing emotions towards an object both vocally and facially, our study used brief videos only displaying torso and arm movements, while facial and vocal information were absent. The lack of facial information, and the unimodality of the presentation might have diminished the effect of social referencing and the impact on infants' manual behavior, which is more energy‐costly than looking, even if the infants still extracted the emotional information from movement kinematics. Thus, this contrast between the looking and touching measures likely reflects the difference in infants' environmental exploration rather than in their emotional development. Future research could compare infants' performance between emotion presentation with movement only (unimodally) or by combining movement with other cues, such as vocal cues. In addition, the stimuli in this study were presented for a pre‐determined length of time, in line with the literature (e.g. Mumme and Fernald 2003), but using movement, which is a fast, dynamic display. This might not have given the infants enough time to fully process the stimuli. Infant‐directed stimulus presentation procedure (e.g. habituation) could be used in future research to ensure infants have enough time to process all the information.

The paradigm used in this study was supposed to allow the infants to visually and manually explore the toys freely and without interference. The infants looked at the toys for over 60% of the trial time (about 20 s), and touched them for over 50% of the trial time (about 17 s), which suggests the paradigm was successful. Moreover, the vast majority of the trials ran until the end, which meant that the attrition rate was low. In contrast to previous research (e.g. Hornik, Risenhoover, and Gunnar 1987; Mumme and Fernald 2003), we included the looking time measure in addition to the touching measure. Although we expected the findings from the two measures to be in line, the looking time was added as a measure of spontaneous preference between the toys, similar to preferential looking paradigms. We also wanted it to serve as a control in case infants did not reach for the toys due to shyness. Indeed, 10 participants did not touch any toy during at least one trial. Thanks to using the looking time measure alongside the playing one, we were able to show that 12‐month‐olds are sensitive to emotional valence in movement kinematics and link the valence with which an object was moved to the object itself, although they do not use it to inform their manual exploration behavior.

Interestingly, this is the first study to use emotional movements expressing fear and disgust, as opposed to anger, used by Addabbo and colleagues (2020) and Schröer and colleagues (2022). As disgust and fear are harder to recognize than happiness, sadness and anger even for older children (Herba et al. 2006), this might have diminished the effect of negative emotional stimuli on infants' behavior. Moreover, whereas the negatively valenced videos were rated as very negative with an average of 1.5 rating on a Likert scale from 0 (negative) to six (positive) by adults, the positively valenced videos were rated as only somewhat positive with an average rating of 3.6. The lower intensity of positively valenced stimuli might have made the differences between the positive and negative emotional valence less salient. However, previous research suggests that the behavioral change in emotion processing tasks is caused by the avoidance of the negatively valenced toys, rather than enhanced interest in the positively valenced toys (Mumme and Fernald 2003). This suggests that provided that the infants were able to perceive the negative emotional valence in the movement, their behavior should not have been changed by perceiving the positive emotional valence. In fact, our results show that the infants did perceive the difference between the emotional valence in movement kinematics of the stimuli, as evidenced by their differential visual exploration.

We find that 12‐month‐old infants can extract the emotional information encoded in movement kinematics using a paradigm where infants can link the information to objects, and freely explore them. Thus, we conceptually replicate Addabbo and colleagues' (2020) findings and extend them to movements performed with fear and disgust as the negative emotions. Moreover, we find that the infants used the emotional information from movement kinematics to inform their visual, but not manual, object exploration. As movement kinematics are only one aspect of emotion expression, future research should help disentangle how they interact with other emotional cues, such as people's voices, faces or body postures.

## Author Contributions

**Joanna M. Rutkowska:** conceptualization, data curation, formal analysis, investigation, methodology, project administration, software, validation, visualization, writing–original draft, writing–review and editing. **Julia Mermier:** conceptualization, investigation, methodology, writing–review and editing. **Marlene Meyer:** conceptualization, methodology, supervision, writing–review and editing. **Hermann Bulf:** conceptualization, funding acquisition, methodology, supervision, writing–review and editing. **Chiara Turati:** conceptualization, funding acquisition, methodology, supervision, writing–review and editing. **Sabine Hunnius:** conceptualization, funding acquisition, methodology, resources, supervision, writing–review and editing.

## Conflicts of Interest

The authors declare no conflicts of interest.

## Supporting information

Supporting Information S1

## Data Availability

The data that support the findings of this study are available in Radboud Data Repository at https://doi.org/10.34973/52ev‐3y73 (Rutkowska et al. 2024).
