# Supplementary material for: Emotional Movement Kinematics Guide Twelve‐Month‐Olds’ Visual, but Not Manual, Exploration
Source: Infancy. 2025 Jan 22;30(1):e70000. doi: 10.1111/infa.70000 (PMC11753196; doi:10.1111/infa.70000)
Supplement: Supplementary file 1 — Supporting Information S1 [file INFA-30-0-s001.docx]

*Supplementary materials*

**Methods**

1. Stimulus creation and stimulus validation

The stimulus videos were newly designed for this study. For the positively valenced stimuli, the actors were instructed to act like they were happy and willing to handle the object, as if it was their favourite toy. For the negatively valenced stimuli, the actors were instructed to act like the object was disgusting (for example like a dirty sock) or scary to them (for example, like a live insect). Each of the three actors recorded several videos moving each of the six toys with positive and negative valence. Out of all recorded videos, 77 videos that were judged best representing each affect (positive/negative) by the researchers were then chosen for a validation study. Each actor provided at least two examples of the action performed with the same valence, for each of the toys (6 toys x 2 emotions x 2 = 24). Actors 1 and 2 contributed 24 videos each, and actor 3 contributed 29 videos. The purpose of this validation study was twofold. Firstly, we wanted to verify that adults are able to discriminate between positive and negative valence in the movements in our stimuli and to label them correctly in an explicit task. Secondly, we wanted to select the most representative videos for each valence to use in the study with infants.

1. Stimulus validation study: Participants, design & procedure

The 77 videos were used in a stimulus validation study conducted online with adults using Gorilla Experiment Builder ([www.gorilla.sc](http://www.gorilla.sc); Anwyl-Irvine et al., 2020). In total, 28 participants (19 female, 8 male, 1 non-binary, mean age M = 24.8 years, SD = 8.4) were recruited via the Radboud University SONA system, and they received 5 euros as compensation for their time. Three participants were excluded from analysis due to not finishing the task (n = 1) and technical problems with the study reported in the comments at the end of the experiment (n = 2). This study was also approved by the local research ethics board (ethical approval code: ECSW2016-0905-396). When the participants signed up for the study, they provided informed consent via an online form and were subsequently redirected to the experimental website. Firstly, they received the task instructions. After each stimulus video was presented to them, they had to first judge whether the emotional valence was positive or negative, then to rate it on a 0 - 6 Likert scale from negative (0), through neutral (3), to positive (6), and finally they had to choose a label for the emotion in the video out of: happiness, sadness, anger, disgust, surprise, and fear (see Supplementary Figure 1).

Figure 1. Procedure of the stimulus validation task in the stimulus validation study with adults.


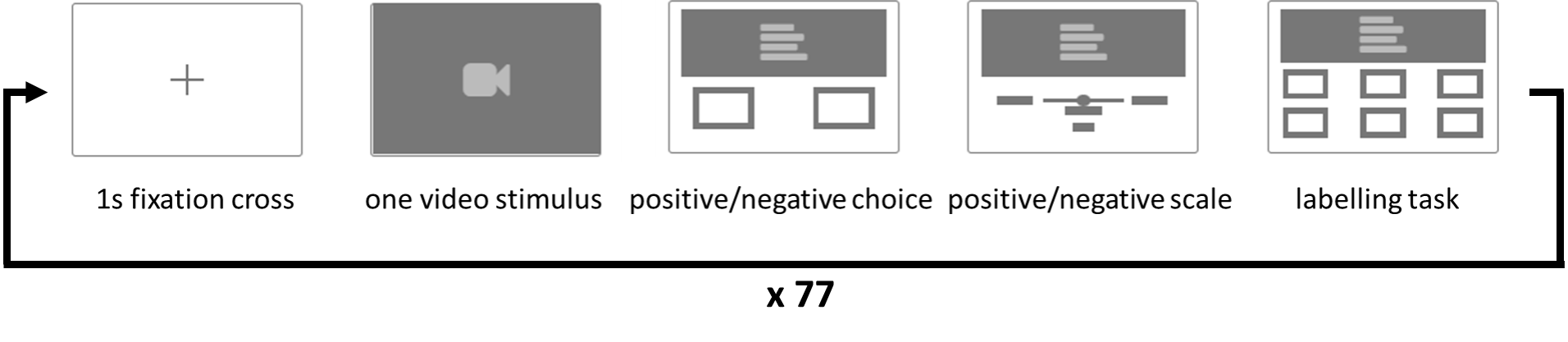


1. Stimulus validation study: Results

From the valence discrimination task, the participants’ and each video’s accuracy was calculated as a proportion of correct responses (i.e. matching with the displayed emotion) out of a total number of responses. The median accuracy for all the videos was 0.88 and it ranged from 0.6 to 1. To measure whether participants performed better than chance on the positive/negative emotion discrimination task, a one-sample Wilcoxon signed rank test was conducted due to non-normal distribution of the data. It revealed that participants’ accuracy (median = 0.9, inter-quartile range = 0.12) was significantly higher than chance, *Z* = 4.38, *p* < 0.001. This means that participants could discriminate between the positive and negative emotion expressed in the stimulus set as a whole, but they found it easier to do so for some videos than others.

From the positive/negative valence rating task, the average rating was extracted for each video. The median rating (0-6 Likert scale) for the positive videos was 3.72 and ranged from 3.36 to 4.44. The median rating for the negative videos was 1.56 and ranged from 1.08 to 2.04. Thus, the videos were rated in line with their emotional valence, that is positive videos on the positive side of the scale (> 3), and negative videos on the negative side of the scale (< 3). From the emotion labelling task, the most chosen emotion label was extracted, and then used for stimulus selection to check if selected stimuli were labelled congruently with the emotional valence.

1. Stimulus selection

Eight out of the 77 videos were selected based on the adult validation study (Rutkowska et al., 2024). At least 2 videos were chosen from each of the three models, one with positive and one with negative emotion. All the chosen videos had to have an accuracy higher than 0.9 on the positive/negative valence discrimination task, and the most frequently chosen label had to be congruent with the valence, that is “happiness” for positive, and “disgust” or “fear” for negative valence (depending on the video). During the selection, it was taken into consideration that the videos featured different toys that would allow for 2 different trial sets and 4 versions of stimulus presentation order (see Supplementary Table 1).

The final stimulus set comprised 8 videos with a mean positive/negative discrimination accuracy of 0.95. The positive videos had a mean rating of 3.61 and the negative videos had a mean rating of 1.54 on the valence scale. All positive videos were most commonly labelled as displaying happiness. Two of the negative emotion videos were most commonly labelled as displaying fear, and two as displaying disgust.

Table 1. Four different orders of stimuli presentation. Each participant was assigned a different version of stimuli order (counter-balanced).

**Version 1**

| Actor | Emotion | Toy colour | Toy shape | Trial |
| --- | --- | --- | --- | --- |
| 2 | negative | green | frog | 1 |
| 2 | positive | orange | duck | 1 |
| 1 | positive | red | octopus | 2 |
| 1 | negative | yellow | duck | 2 |

**Version 2**

| Actor | Emotion | Toy colour | Toy shape | Trial |
| --- | --- | --- | --- | --- |
| 1 | positive | red | octopus | 1 |
| 1 | negative | yellow | duck | 1 |
| 2 | negative | green | frog | 2 |
| 2 | positive | orange | duck | 2 |

**Version 3**

| Actor | Emotion | Toy colour | Toy shape | Trial |
| --- | --- | --- | --- | --- |
| 3 | positive | green | turtle | 1 |
| 3 | negative | red | octopus | 1 |
| 1 | negative | orange | duck | 2 |
| 1 | positive | yellow | frog | 2 |

**Version 4**

| Actor | Emotion | Toy colour | Toy shape | Trial |
| --- | --- | --- | --- | --- |
| 1 | negative | orange | duck | 1 |
| 1 | positive | yellow | frog | 1 |
| 3 | positive | green | turtle | 2 |
| 3 | negative | red | octopus | 2 |

**Results**

**Supplementary plots for the Bayesian generalised linear mixed models**

Figure 2. Trace and Density Plots for the Bayesian generalised linear mixed model predicting the proportion of looking towards a toy from its emotional valence (positive/negative) and trial (1/2) with subject as a random effect.


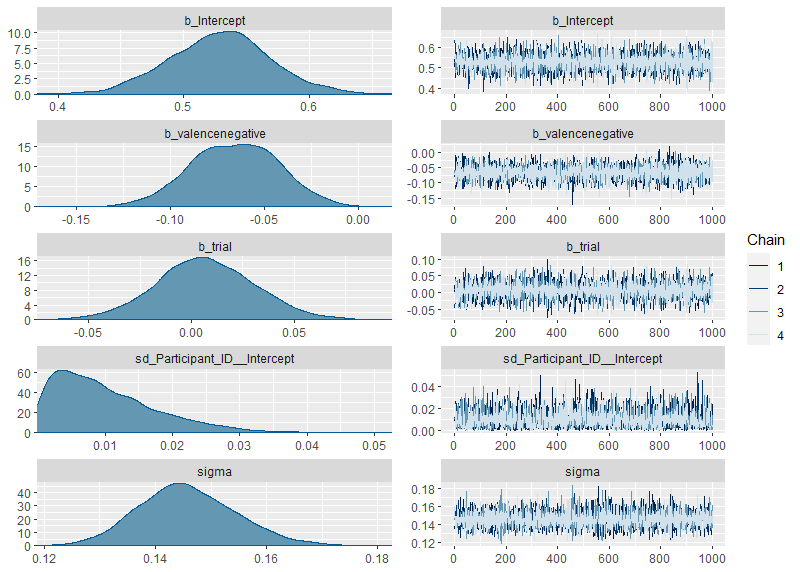


Figure 3. Trace and Density Plots for the Bayesian generalised linear mixed model predicting the proportion of looking towards a toy from its emotional valence (positive/negative) and trial (1/2) with subject as a random effect.


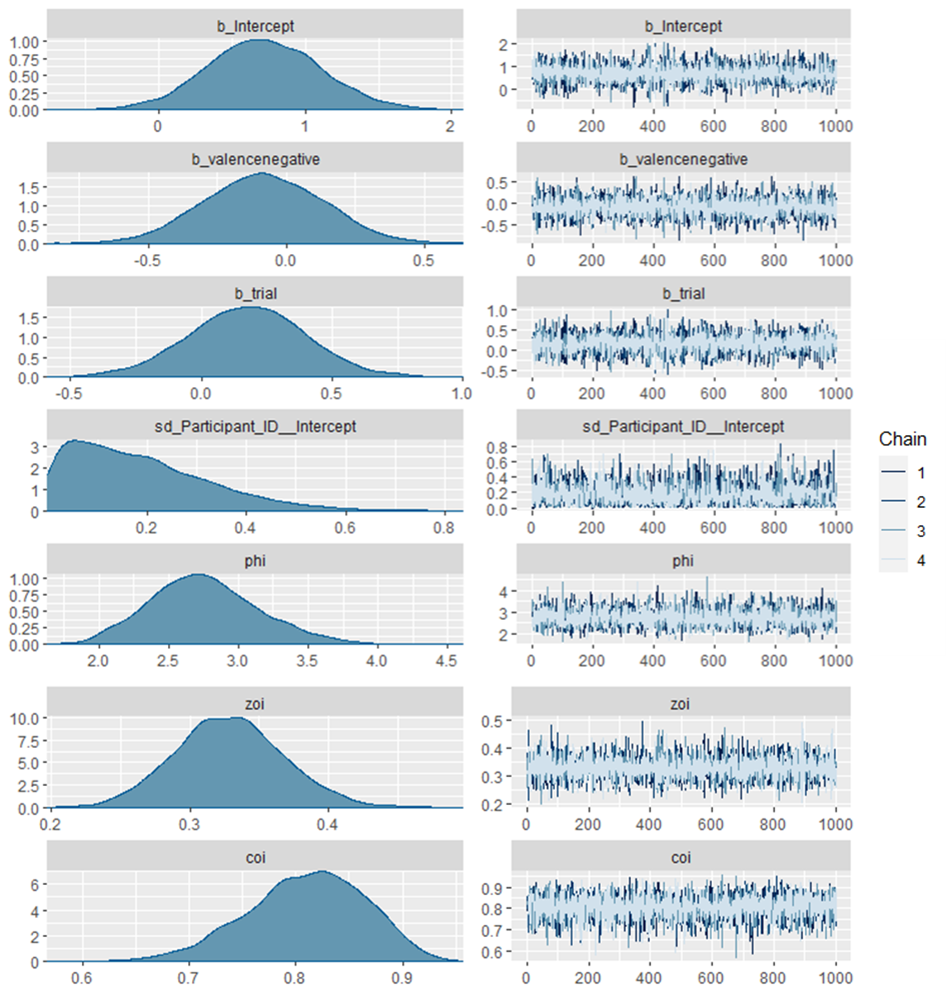


**Supplementary analysis**

*Relationship between the proportion of looking and touching*

In this analysis, the proportions of looking and touching for positive and negative toys were separately averaged between two trials if an infant completed both test trials, and touched at least one of the toys on both trials, or taken from the one trial completed by an infant in which they have touched at least one toy (n = 48). Due to the nature of the data, i.e. some proportion values equal to 0, it was not possible to compute a ratio between touching the positively and negatively valenced toys. In addition, there is a strong relationship between the proportions of looking and touching between the positive and negative toys, so they had to be analysed separately.

Two two-tailed Bayesian Kendall’s tau-b correlations were conducted in JASP to examine the relationship between infants’ touching and looking at the toys presented with the same emotional valence (positive/negative) with a default stretched beta prior with the width of 1. We decided for the non-parametric correlation analysis due to the non-normal distribution of the touching data.

Two two-tailed Bayesian Kendall’s tau-b correlations revealed no evidence for or against the relationship between the touching and looking at toys presented with negative valence (BF10 = 1, r_τ_ = 0.19, 89% Credible Intervals CI [0.022, 0.326]; see Figure 4), and moderate evidence for no relationship between the touching and looking at toys presented with positive valence (BF01= 4.7, r_τ_ = 0.05, 89% CI [-0.103, 0.202]; see Figure 5). Thus, there was no evidence for a relationship between infants’ looking and touching in our task.

Figure 4. (a) A scatterplot of the relationship between the ranked proportions of looking and touching of the toy presented with negative valence. (b) A prior and posterior distribution of Kendall’s τ (tau) with 89% Credible Interval for the Kendall’s tau-b correlation between the average touching and looking at toys presented with negative valence, showing possible effect values. The plot shows the estimate of Kendall’s τ (tau) after updating prior knowledge with data. The grey dots indicate the density values of the prior and posterior distributions at test value. The dot being higher on the posterior than on the prior distribution shows the evidence for null hypothesis. (c) A robustness plot showing the BF as a function of the scaling factor of the prior.


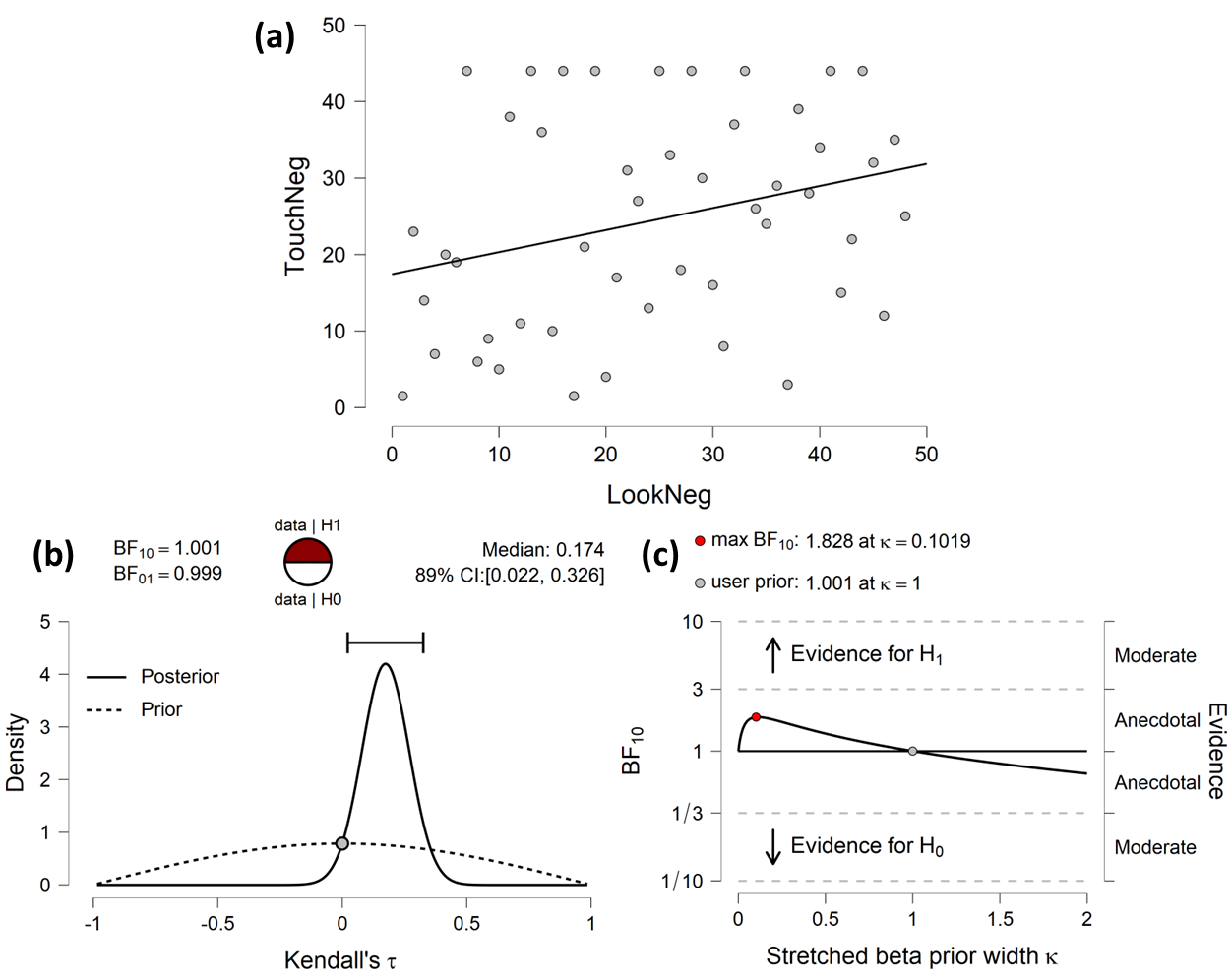


Figure 5. (a) A scatterplot of the relationship between the ranked proportions of looking and touching of the toy presented with positive valence. (b) A prior and posterior distribution of Kendall’s τ (tau) with 89% Credible Interval for the Kendall’s tau-b correlation between the average touching and looking at toys presented with positive valence, showing possible effect values. The plot shows the estimate of Kendall’s τ (tau) after updating prior knowledge with data. The grey dots indicate the density values of the prior and posterior distributions at test value. The dot being higher on the posterior than on the prior distribution shows the evidence for null hypothesis. (c) A robustness plot showing the BF as a function of the scaling factor of the prior.


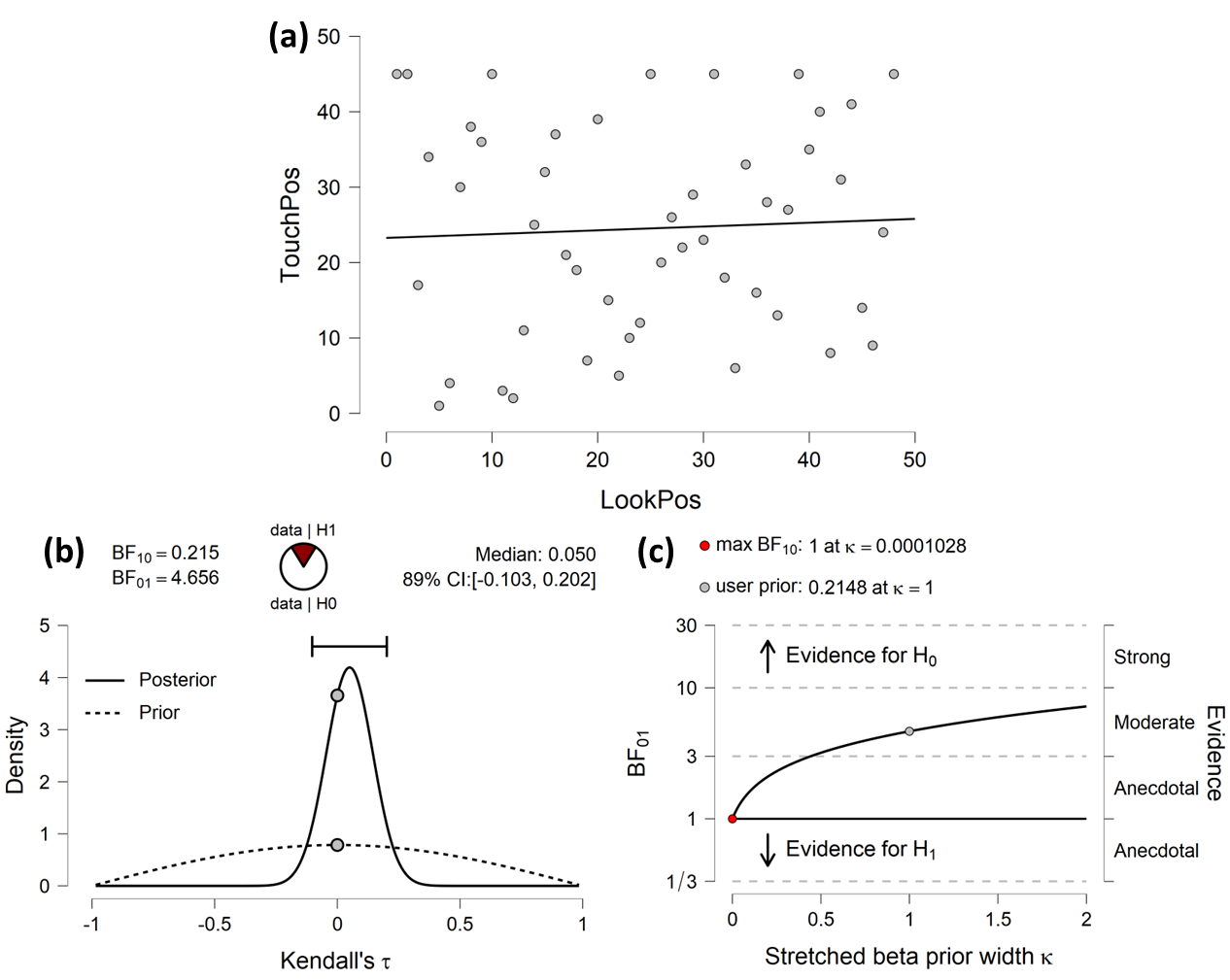


*Relationship between first touch and looking preference*

For each test trial, infants were divided into 2 groups based on their first touch: the ones that touched the toys presented with positive emotional valence, and the ones that touched the toys presented with negative emotional valence. In each trial, their preference towards looking at the positive toy was calculated by dividing the proportion of looking at the positive toy by the proportion of looking at the negative toy. The looking preference variable was log-transformed for further analysis due to non-normal distribution of the data. One outlier was removed from the data (looking preference = 10.82).

We fitted a Bayesian generalised linear mixed model using the *brms* package in R to predict the preference to look at the positive toy from infants’ first touch (positive/negative) and trial (1/2) with subject as a random effect.

The effect of positive touch has a probability of 76.5% of being positive (Median = 0.05, 89% CI [-0.06, 0.16], and can be considered of undecided significance [27% in ROPE] (see Figure 6). The effect of trial has a probability of 57% of being negative (Median = -0.01, 89% CI [-0.11, 0.09]) and can be considered of undecided significance [36% in ROPE]. Thus, we did not find evidence for the difference in looking preference between children that touched positive and negative toys first (see Figure 7).

Figure 6. Trace and Density Plots for the Bayesian generalised linear mixed model predicting the looking preference at the positive toy from infants’ first touch (positive/negative and trial (1/2) with subject as a random effect.


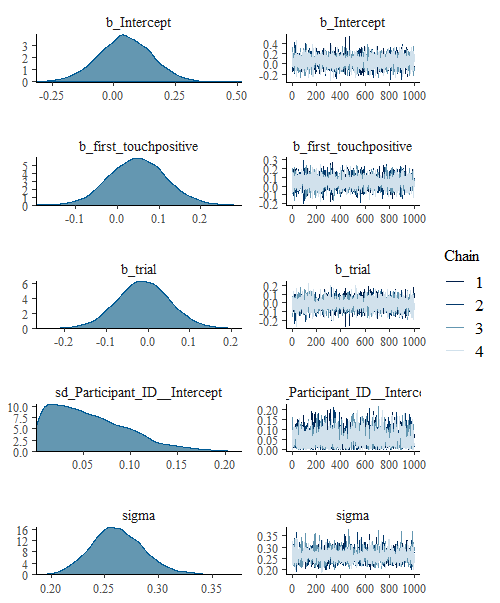


Figure 7. A pirate plot of the looking preference at the positive toy of infants who touched positive and negative toys first in both trials; each dot represents a data point, the bold black horizontal line represents the mean of each condition, and the semi-transparent box shows the 89% highest density interval (HDI) around the mean.


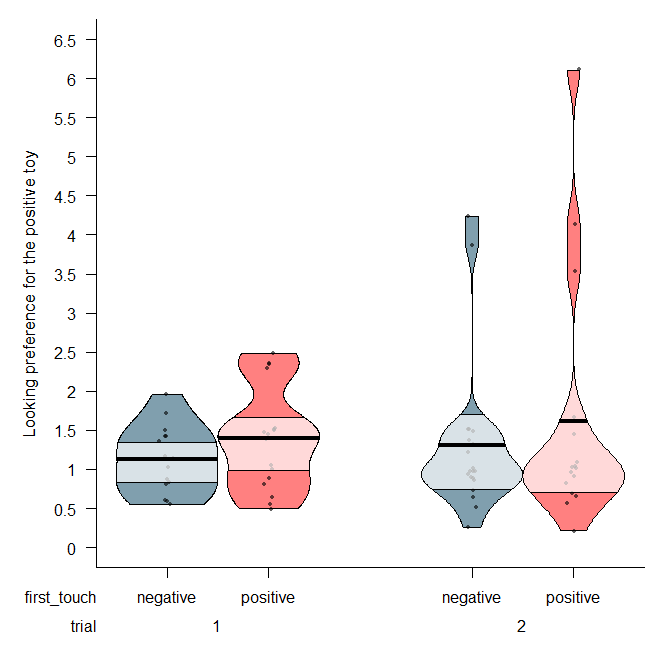


**References**

Rutkowska, J.M., Mermier, J., Meyer, M., Bulf, H., Turati, C., & Hunnius, S. (2024). Emotional movement kinematics guide twelve-month-olds’ visual, but not manual, exploration (Radboud University, Version 1) [Data set]. Radboud University. <https://doi.org/10.34973/52ev-3y73>
